# Expression Profiling of Rectal Tumors Defines Response to Neoadjuvant Treatment Related Genes

**DOI:** 10.1371/journal.pone.0112189

**Published:** 2014-11-07

**Authors:** Pablo Palma, Carlos Cano, Raquel Conde-Muiño, Ana Comino, Pablo Bueno, J. Antonio Ferrón, Marta Cuadros

**Affiliations:** 1 Division of Colon & Rectal Surgery, Virgen de las Nieves Hospital (HUVN), Granada, Spain; 2 Department of Computer Science and Artificial Intelligence, University of Granada, Granada, Spain; 3 Department of Surgical Research, Virgen de las Nieves Hospital (HUVN), Granada, Spain; 4 Department of Biochemistry and Molecular Biology III e Immunology, University of Granada, Granada, Spain; 5 GENYO, Centre for Genomics and Oncological Research: Pfizer/University of de Granada/Junta de Andalucía, Granada, Spain; Heidelberg University, Germany

## Abstract

To date, no effective method exists that predicts the response to preoperative chemoradiation (CRT) in locally advanced rectal cancer (LARC). Nevertheless, identification of patients who have a higher likelihood of responding to preoperative CRT could be crucial in decreasing treatment morbidity and avoiding expensive and time-consuming treatments. The aim of this study was to identify signatures or molecular markers related to response to pre-operative CRT in LARC. We analyzed the gene expression profiles of 26 pre-treatment biopsies of LARC (10 responders and 16 non-responders) without metastasis using Human WG CodeLink microarray platform. Two hundred and fifty seven genes were differentially over-expressed in the responder patient subgroup. Ingenuity Pathway Analysis revealed a significant ratio of differentially expressed genes related to cancer, cellular growth and proliferation pathways, and c-Myc network. We demonstrated that high Gng4, c-Myc, Pola1, and Rrm1 mRNA expression levels was a significant prognostic factor for response to treatment in LARC patients (p<0.05). Using this gene set, we were able to establish a new model for predicting the response to CRT in rectal cancer with a sensitivity of 60% and 100% specificity. Our results reflect the value of gene expression profiling to gain insight about the molecular pathways involved in the response to treatment of LARC patients. These findings could be clinically relevant and support the use of mRNA levels when aiming to identify patients who respond to CRT therapy.

## Introduction

There has been a high local recurrence rate in locally advanced rectal cancer (LARC). Besides improvements in surgical techniques, both neoadjuvant short-course radiotherapy and long-course chemoradiation (CRT) improve oncological results [1]. After CRT, the ability to achieve tumor reduction or even a complete response is observed in up to 60% of the patients treated. This treatment also correlates with a decreasing local recurrence. Conversely patients with a poor response have a worse oncological outcome.

To date, there is no effective method of predicting the response to CRT and therefore such aggressive schedule is indicated for all patients with LARC [2]. Nevertheless, identification of patients who have a higher likelihood of responding to preoperative CRT could be crucial in decreasing treatment morbidity and avoiding expensive and time-consuming treatments [3]. There are undoubtedly many patient and tumor factors contributing to tumor response. Genetic and molecular profiling of rectal tumours has provided an insight into tumor biology. Gene expression profiling has been extensively applied to study colorectal tumors, and gene signatures for recurrence, prognosis or even response to chemotherapy have been described [4]. Nevertheless, to date, none of the identified signatures or molecular markers in LARC has been successfully validated as a diagnostic or prognostic tool applicable to routine clinical practice. We explored whether tumoral tissue transcriptional profiling might unveil signatures indicative of response to preoperative CRT.

## Material and Methods

### Study cohort

The study was approved by the Ethics Committee (CEI Granada), Department of Health, Government of Andalucía, Spain. Participants provided written consent in accordance with institutional and national guidelines; consent procedure was also approved by the Ethics Committee.

The microarrays study included a total of 35 consecutive enrolled patients with LARC treated at our Division of Colon & Rectal Surgery of the Virgen de las Nieves University Hospital with additional 8 patients in the validation group.

The inclusion criteria were: histologically proven rectal tumor at a clinical stage II-III (cT3-4/and or N positive) following endorectal ultrasound and/or MRI scan. Patients were excluded if they had tumor located above 13 cm from the anal verge by rigid rectoscopy, colonic cancer assessed by colonoscopy, distant metastases by abdominal and thoracic PET-CT scan, and suspicion of hereditary colorectal cancer.

Pretherapeutic staging was performed, including complete medical history and physical evaluation, digital rectal examination, endorectal ultrasound, rigid rectoscopy, colonoscopy, PET-TC and MRI. Tumor samples were prospectively obtained upon rectoscopy. All patients included subsequently received a total dose of 50.4Gy of radiation (28 fractions of 1.8Gy) associated with capecitabine alone or capecitabine combined with oxaliplatine, according to our Hospital Clinical Practice Guidelines. Standardized surgery was performed, including total mesorectal excision, after an interval of 8 weeks after CRT.

The tumor response was assessed in surgical specimens by pathological examination based on the semiquantitative tumor regression grading (TRG) system described by Mandard in 1994 [5]: TRG1 and TRG2 scores were considered responders, whereas TRG3, TRG4, and TRG5 scores were classified as non-responders.

### RNA isolation and microarray analyses

Frozen sample materials were provided by the Tissue and Tumor Bank, Department of Pathology at the Virgen de las Nieves Hospital. RNA was extracted from macrodissected frozen samples according to standard procedures using RNesasy minikit (Qiagen Sciences). RNA quantity and integrity were checked by spectrophotometry in a NanoDrop (ND-1000, DE, USA) and in an Experion automated electrophoresis system (Bio-Rad, Richmond, VI, USA), respectively. Prior to extraction, 8 µm sections were stained with hematoxylin and eosin for microscopic examination to check the percentage of tumor cells. The percentage of tumor cells was estimated by an experienced pathologist in each case via visual inspection.

Microarrays were done in duplicate using 10 µg of cRNA. After reverse transcription, cRNAs were labeled with Cy5 streptavidine. Hybridization of 20,000 human genes CodeLink bioarrays (Applied Microarrays, Tempe, Ariz, USA) was performed overnight at 37°C in a shaker. Microarrays were read with a GenePix 4000B laser scanner (Axon Instruments, CA), quantified, and normalized with CodeLink Software 4.2 (Applied Microarrays, Tempe, AZ).

Microarray data were normalized using different normalization methods: average normalization and cyclic loess [6]. The quality of the outcome was assessed by different plots produced by the software package ArrayQualityMetrics implemented in the R language. Samples were grouped Responders and Non-Responders, and the differential expression of genes was then evaluated using the software SAM (Significance Analysis of Microarrays, Stanford University, Stanford, CA). Genes with p-values <0.05 were considered as significantly differentially expressed between the two subgroups. This set of genes constitutes our molecular signature to predict response to treatment after CRT.

Raw and normalized gene expression values for each sample under study have been made publicly available at the Gene Expression Omnibus GEO database with submission number GSE53781.

### Functional analysis of gene expression results

To obtain information about the biological signature and to analyze the biological coherence of the microarray results, gene expression data were analyzed using Ingenuity Pathways Analysis (IPA) v.5.0 (Ingenuity Systems Inc, Redwood City, CA). This tool provides information about diseases, molecular function and biological process categories, as well as biological pathways related to the genes obtained from the microarray analysis. Ingenuity Pathways Analysis was also used to identify potential biomarkers. In addition, IPA maps each gene within a global molecular network developed from information contained in the Ingenuity Pathways Knowledge Base. Gene networks are generated algorithmically based on their connectivity in terms of expression, activation, transcription, and/or inhibition. A network in IPA is defined as a graphical representation of the molecular relationships between genes, represented with nodes, and the biological relationship between them represented by connecting lines. All connections are supported by published data stored in the Ingenuity Pathways Knowledge Base and/or PubMed. IPA ranks all genes based on their connectivity, using a generalization of the concept of node degree, which measures the number of single genes to which a gene is connected.

### Quantitative Reverse Transcription-PCR analysis (qRT-PCR)

To validate microarray experimental data, we determined the level of expression of 20 genes in rectal tumor patients by real-time quantitative reverse transcription-PCR. In addition, to validate selected candidate genes, an independent series of 8 consecutive LARC was analyzed using qRT-PCR.

The genes Abcb7, Cd81, Chmp4b, Cri2, Ect2, Ska2, Gng4, Id1, Mmp12, c-Myc, Nat5, Rrm1, Rfsbp1, Stmn1, Stmn2, Top1mt, Mapk9, P53csv, Dpm1, and Pola1, were selected for this validation among the obtained set of genes that discriminates Responders vs. Non-Responders. In addition, the tumor protein 53 (Tp53) and the cyclin-dependent kinase inhibitor 1a (Cdkn1a) were also analyzed due to their relation to colorectal cancer. We optimized a sensitive and specific qRT-PCR assay using MX3005P QPCR System (Agilent Technologies, Santa Clara, CA). One microgram of RNA was used for reverse transcription with QPCR-grade AffinityScript Multiple Temperature Reverse Transcriptase (AffinityScript QPCR cDNA, Agilent Stratagene) using random hexamers. PCR reactions contained 1 µg cDNA, 12.5 µL qPCR Master Mix, 12.5 uL of solaris qPCR master mix, 1.25 uL of solaris primer/probe set for each gene. PCR conditions were 15 min at 95°C, 15 s at 95°C and 1 min at 60°C for 40 cycles. We designed specific Taqman probes and primers for each gene (**Table S1**). The cycle threshold (Ct, the PCR cycle at which probe signal reaches the threshold) was determined for each gene. Before performing this study, Gapdh, Rpl13a, and Tbp were selected as candidate housekeeping genes. Gapdh emerged as the most stable gene with no closely comparable housekeeping gene among the evaluated genes in the series of tumors. Expression was quantified following the analysis of two different dilutions of cDNAs (1 and 1/10) in triplicate. For each experimental sample, the amount of each gene and endogenous reference (Gapdh) was determined from the standard curves. These standard curves were composed of five points obtained from five-fold serial dilutions (1, 1/10, 1/50, 1/100, and 1/500) of cDNA from Universal Human Reference RNA (Stratagene). This cDNA is composed of total RNA from 10 human cell lines. We considered only experiments in which the linear relationship between Ct (threshold cycle) and the log of the amount of standard curve for each gen and, Gapdh were higher than 0.99 (correlation coefficient). The average Ct of the triplicates was calculated, excluding outliers (replicates with Ct differing by more than one cycle from the median). If the sample failed to meet these criteria a third time, it was classified as an assay failure. The expression values of each gene were then divided by the amount of Gapdh to obtain a normalized value. Gapdh gene was used as an internal control for RNA quality reverse transcription and to correct the variations in the degree of RNA degradation.

### Fluorescence in situ hybridization (FISH)

Paraffin-embedded pre-treatment core biopsies from patients with LARC (7 Responders and 6 Non Responders) were tested for c-Myc amplification. For the detection of c-Myc amplification we used the c-Myc dual fusion break-apart (Dako, Santa Clara, CA; Y5410) and CEP 8 (Dako 30-170008) probes, and followed the manufacturer's recommendations.

The c-Myc dual-fusion probe consists of pairs of probes labeled in distinct colors (red and green), with each probe binding to a different part of the gene. The FISH DNA probes are a mixture of a Texas Red-labeled DNA probe (c-Myc-downstream) covering 418 kb telomeric to the c-Myc breakpoint cluster region and a fluorescein-labeled DNA probe (c-Myc-upstream) covering 652 kb centromeric to the c-Myc breakpoint cluster region. In a normal intact cell, two separate red and two separate green individual signals will be visible, whereas an altered pattern of c-Myc would generate two fused red/green signals (often appearing as single yellow signals), accompanied by one red and one green signal (representing the normal loci). To identify and enumerate chromosome 8, we used the centromere 8 (CEP8) DNA probe that detects rich alpha satellite sequences in the centromere region of chromosome 8.

The FISH testing methodology is a semiquantitative method based on the computation of the average ratio of c-Myc signals to CEP8 signals in non-overlapping interphase nuclei of the lesion. Tumors with a c-Myc: CEP8 ratio > = 2∶1 were considered positive for gene amplification. The c-Myc and CEP8 signals were visualized by two blinded and independent pathologists.

All cases without a consensus diagnosis were reviewed jointly on a multiheaded microscope and discussed by the two blinded expert pathologists.

### Immunohistochemical analysis

Immunohistochemistry with antibody against c-Myc was performed. 4 µm thick in formalin-fixed paraffin-embedded tissue (FFPE) full tissue sections were stained for c-MYC (rabbit monoclonal anti-human c-MYC antibody; catalog #1472-1, Epitomics, Inc., Burlingame, CA) in the Specialized Histopathology Laboratory and the Anatomic Pathology Immunohistochemistry Laboratory (Spanish National Cancer Centre) on Ventana Benchmark XTs (Ventana Medical Systems, Tucson, AZ) using extended antigen retrieval (CC1 buffer), anti-c-MYC antibody (final concentration 0.56 µg/mL) and signal amplification (mouse anti-rabbit reagent followed by rabbit anti-mouse reagent).

The percentage of positive tumor nuclei was manually scored from 0 to 100% in 10% intervals. Independent scoring by two blinded expert pathologists showed concordance for final c-Myc score.

### Statistical analysis

To determine differences in clinicopathological features between response and non-response patients, Student t-test was used to compare means of continuous variables, and Chi-square or 2-sided Fisher exact test were chosen for categorical variables.

Statistical significance of differences in transcript levels was assessed using the non-parametric T-test (Mann Whitney).

Data analyses were carried out with the SPSS statistical software, version 15.0 (SPSS Inc., Chicago, IL). Sample size was calculated to obtain a power of 0.8.

## Results

### Patient and tumor characteristic

Nine of the initial 35 patients were excluded due to the poor quality of the RNA or contradictory results of Mandard's criteria and histopathological downstaging. Complete clinical data regarding, age, sex, stage of disease, response to therapy, and overall survival from 26 patients (10 responders and 16 non-responders) is shown in **Table 1**. The patient cohort was homogeneous, no statistically significant differences were found in terms of CRT, surgery or sex when comparing the two groups (response vs. non-response) (**Table 2**). The main characteristics of the 8 patients included in the validation set are shown in **Table 1**.

**Table 1:** Characteristic of locally advanced rectal cancer patients included in this study.

		Sex	Age	CRT	cTN	Surg	TRG	Downst	Downs	Resp	CPR
Initial cohort	1	M	63	Capox	T4N1	LAR	2	YES	YES	YES	NO
	2	M	71	Cape	T3N0	LAR	2	YES	YES	YES	NO
	3	M	77	Cape	T3N1	LAR	4	YES	NO	NO	NO
	4	M	67	Cape	T3N0	LAR	5	NO	NO	NO	NO
	5	W	83	Cape	T3NO	APR	2	YES	YES	YES	NO
	6	W	63	Cape	T3N2	LAR	5	NO	YES	NO	NO
	7	M	53	Capox	T3N1	LAR	1	YES	YES	YES	YES
	8	M	64	Capox	T3N2	HART	2	NO	YES	YES	NO
	9	M	69	Cape	T3N0	HART	3	YES	YES	NO	NO
	10	M	69	Cape	T3N0	LAR	1	YES	YES	YES	YES
	11	M	71	Cape	T3N0	LAR	5	YES	YES	NO	NO
	12	W	62	Cape	T3N1	HART	5	YES	NO	NO	NO
	13	W	58	Cape	T3N1	LAR	1	YES	YES	YES	YES
	14	M	50	Capox	T4N0	APR	4	NO	NO	NO	NO
	15	M	36	Capox	T4N0	HART	5	NO	NO	NO	NO
	16	M	54	Cape	T3N0	LAR	4	YES	YES	NO	NO
	17	M	47	Capox	T3N0	LAR	4	NO	NO	NO	NO
	18	M	45	Capox	T3N0	APR	5	NO	NO	NO	NO
	19	M	47	Capox	T3N1	HART	1	YES	YES	YES	YES
	20	M	74	Cape	T3N0	HART	4	YES	YES	NO	NO
	21	W	61	Cape	T3N1	LAR	4	NO	YES	NO	NO
	22	W	37	Capox	T3N2	LAR	5	NO	NO	NO	NO
	23	M	54	Cape	T3N0	LAR	1	YES	YES	YES	YES
	24	M	69	Capox	T3N2	APR	3	NO	NO	NO	NO
	25	W	70	Cape	T3N2	LAR	3	YES	NO	NO	NO
	26	M	61	Capox	T3N1	LAR	2	YES	YES	YES	NO
Validation cohort	27	W	76	Cape	T3N0	LAR	4	YES	YES	NO	NO
	28	W	64	Cape	T3N2	LAR	4	NO	NO	NO	NO
	29	M	63	Cape	T3N1	LAR	2	YES	YES	YES	NO
	30	W	56	Cape	T3N1	LAR	3	YES	NO	NO	NO
	31	M	62	Cape	T3N1	LAR	4	NO	NO	NO	NO
	32	M	64	Cape	T3N2	LAR	3	NO	NO	NO	NO
	33	M	56	Cape	T4N1	LAR	3	YES	YES	NO	NO
	34	M	62	Cape	T3N1	LAR	4	NO	NO	NO	NO

**Table 2:** Patient characteristic stratified by response to treatment.

	Responders	Non responders	p
**Sex**			0.668
Woman	2 (20%)	5 (31.3%)	
Man	8 (80%)	11 (68.7%)	
**Age** (mean values ± standard deviation)	63.2±3.5	59.5±3.2	0.473
**Chemotherapy**			0.689
Capecitabine	5 (50%)	10 (62.5%)	
Capecitabine + oxaliplatine	5 (50%)	6 (37.5%)	
**Surgical technique**			1.000
Low anterior resection	9 (90%)	13 (81.2%)	
Abdmino-perineal resection	1 (10%)	3 (18.8%)	

### Differential gene expression between treatment responder and non responder rectal tumor patients

A supervised method (Significance Analysis of Microarrays -SAM-) was used to find statistically significant (adjusted p<0.05) differentially expressed genes between treatment responder and non-responder LARC patients. We found 260 clones, representing 257 genes (**Table S2**), that were differentially expressed between these two subgroups. All of them presented significantly higher expression levels in responder LARC samples. A hierarchical clustering analysis was then performed on both the genes and the samples based on the expression values of these 257 genes in the 26 rectal tumoral samples (**Figure S1**). Samples were clustered into 2 main subgroups (branches). One of the subgroups contained half of the responder tumor samples, and they clearly showed over-expression of these genes, while the other subgroup was characterized by lower expression of them. The other branch of samples contained the remaining responder samples, and non responder samples. This subgroup also contained four branches; in one of them 40% of responder tumor patients were grouped together. Differences in expression of the genes between responder and non responder rectal tumor samples cannot be explained by a different content of tumor cells within the samples since the estimation of tumor cells in the tumor samples showed no significant differences between these two subgroups (data not shown).

**Figure 1:**
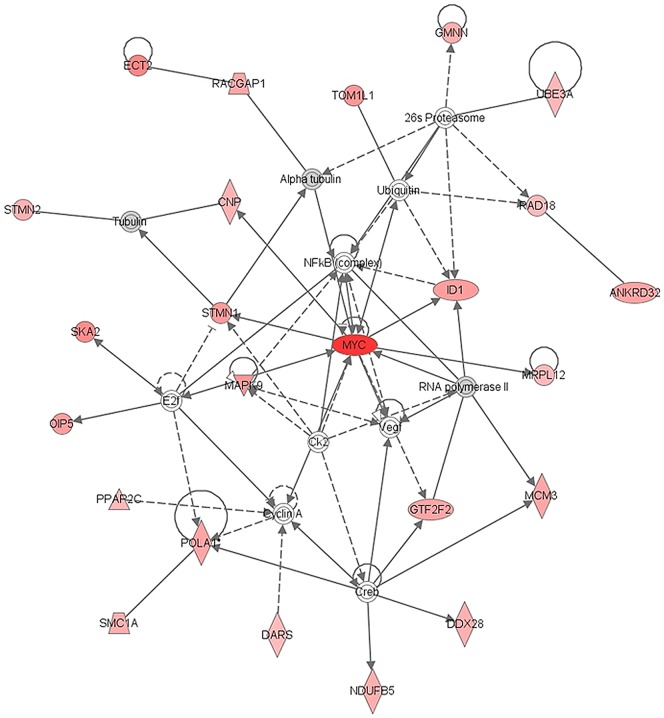
IPA's Key regulatory network over-expressed in responder patients. A network is a graphical representation of the relationships between molecules. Molecules are represented as nodes, and the biological relationship between 2 Ingenuity nodes is represented as an edge (line). All edges are supported by at least 1 reference from the literature, from a textbook, or from canonical information stored in the Ingenuity Knowledge Base. Network analysis analyses identified two major inducers: c-Myc and Pola1.

In order to better understand the biological meaning of the genes showing higher expression levels in responder rectal tumor patients, data from these 257 genes were analysed using the IPA software to enable the identification of interacting genes within our networks that were not part of our focus gene lists. An analysis of the identified genes (p<0.05) showed that the encoding proteins associated with several canonical pathways, such as *Pyrimidine* and *Purine Metabolism* (p = 0.022), and *Colorectal Cancer Metastasis Signalling* (p = 0.02). In the diseases and disorders, and molecular and cellular function categories, most of these genes were related to *Cancer* (p<0.001), and *Cellular Growth and Proliferation* (p<0.001), involving 33 and 19 molecules respectively. IPA software was also used to build up networks that involved the selected 257 genes. The most significant IPA network consisting of 49 genes, contained 24 focus genes (Nkrd32, Cnp, Dars, Ddx28, Ect2, Gmnn, Gtf2f2, Id1, Mapk9, Mcm3, Mrpl12, Myc, Ndufb5, Oip5, Pola1, Ppap2c, Racgap1, Rad18, Ska2, Smc1a, Stmn1, Stmn2, Tom1l1, and Ube3a) with direct or indirect connections related to gene expression, protein-DNA and protein-protein interactions. These genes were directly or indirectly connected to a c-Myc network. The c-Myc network, in which red-labelled symbols indicate 24 genes contained in our list of 257 genes, is shown in **Figure 1**.

### Real-Time Reverse Transcriptase (RT)-PCR Validation of Microarray Observations

To confirm the findings obtained in the expression array, qRT-PCR was performed for selected genes in 24 LARC samples: responder (n = 10) and non responder (n = 14). Samples of 2 patients (6 and 9) were not analyzed because of poor RNA quality. Among the genes of the c-Myc apoptosis pathway, c-Myc, Mapk9 (one of the main kinases involved in the phosphorylation of c-Myc), and the Id1 and Stmn1 genes, downstream targets of c-Myc, were selected to analyze their expression by real-time RT-PCR. In addition, we analysed other genes related to c-Myc apoptosis and proliferation (Tp53, P53csv, Pola1, Cdkn1a, Top1mt, Rrm1, and Ska2), as well as genes involved in angiogenesis (Mmp12), and transport (Abcb7, and Chmp4b). Highly concordant results were obtained for all these genes with statistically significant differences between these two groups of LARC patients (**Figure S2**).

### Validation of predictive biomarkers

For the genes Gng4, c-Myc, Pola1 and Rrm1 (a subset of the gene signature obtained by the microarray analyses) we computed receiver operating characteristic (ROC) curves. Area under curve (AUC) values and 95% confidence intervals (CI) were calculated to determine the specificity and sensitivity of response to treatment prediction. ROC curves of Gng4, c-Myc, Pola1 and Rrm1 microarray data reflected the ability to distinguish between the responder subgroup and non-responder subgroup, with an AUC of 0.750, 0.862, 0.850 and 0.806, respectively. For Gng4 the cut-off point set at 5.59 yielded a sensitivity of 70% and a specificity of 81.3%. At a cut-off point set at 64.45 for c-Myc yielded a sensitivity of 70% and a specificity of 100%. At a cut-off point set for Pola1 at 167.64 yielded a sensitivity of 60% and a specificity of 87.5%. At a cut-off point set at 5.52 for Rrm1 yielded a sensitivity of 60% and a specificity of 75%. For best result, the ROC curve was generated with data from c-Myc qRT-PCR. Sensitivity (60%) and specificity (80%) were worse with an AUC value of 0.733 (all data available in **Table S3**).

We then assessed the power of the selected genetic signature (Gng4, c-Myc, Pola1 and Rrm1) to predict response to therapy in LARC. We considered a test positive when at least 3 of the 4 genes were over-expressed in the sample under study. The genetic signature achieved 60% sensitivity, 100% specificity, and 85% accuracy to identify responder patients.

### Gen amplification (rewrite headline)

**Figure 2:**
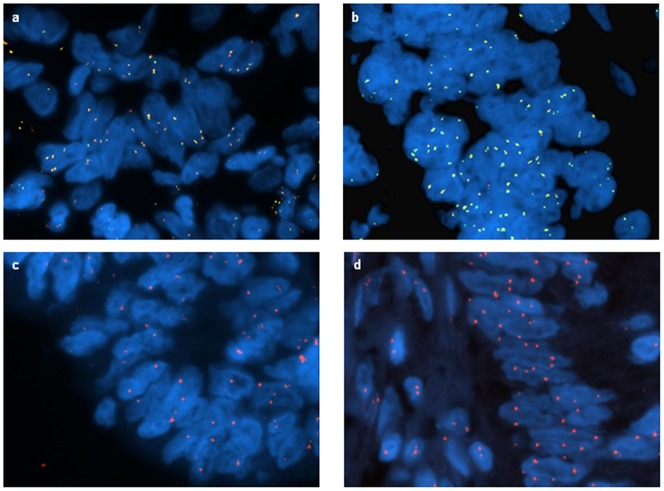
Representative fluorescence in situ hybridization (FISH) signal patterns using the c-Myc break-apart and CEP8 probe in locally advanced rectal cancer (LARC); a, b) c-Myc rearrangement is absent as evidenced by the presence of normal red-green fusion signals only. Multiple copies (3–4 copies or 4–6 copies) of c-Myc are present in the tumor; c, d) Two copies of chromosome 8.

Fluorescence in situ hybridization (FISH) was applied to LARC for the detection of c-Myc amplification, and the results were compared with expression levels of c-Myc mRNA. Successful probe hybridization was achieved in 13 cases (7 responders and 6 non-responders). Representative results of FISH examination of c-Myc are shown in **Figure 2**. We found an altered pattern of c-Myc in 5 of 13 tumors (38%): 4 Responder and 1 Non-Responder LARC samples; however, they also had two number of centromere 8 signals. Amplification of c-Myc was not seen in any studied cased with LARC, suggesting that overexpression of c-Myc can also occur via mechanisms independent of gene amplification.

### c-Myc detection for immunohistochemistry

Immunohistochemical analysis was also performed for c-Myc to study whether the differences in the expression detected by microarray analysis could also be detected at a protein level.

IHC analysis of 12 pre-treatment LARC (6 Responder and 6 Non-Responder) cases revealed a spectrum of total tumor cell nuclei (ranging from 90% to100%) that stained positive for c-Myc by manual scoring. Responder tumors exhibited strong c-Myc staining intensity. Non tumoral cells within the tumor did not express c-Myc protein.

The study of c-Myc activation based on the expression of c-Myc protein did not reveal differences in the intensity of this protein in the different patient subgroups (responder versus non-responder), suggesting that post-translational modifications, as well as the half-live or abundance might affect the relationships between the two data types.

## Discussion

Gene expression profiling using microarray technology has led to a series of promising results though tissue gene expression profiling of different malignancies, including cancer. Nevertheless, to date, none of the identified signatures or molecular markers in LARC has been successfully validated as a diagnostic or prognostic tool applicable to routine clinical practice. Moreover, there has been little agreement between signatures, with scarce overlap in the reported genes [4]–[16]. Only two genes, MMP4 and FLNA, have been reported in more than one paper [7], [8], [13] and one of the 257 genes reported in this study RRM1 (an important marker for chemotherapy resistance in colon tumors [17]), was also identified by Nishioka [13]. This could be due to variations in the inclusions criteria, schedule of neoadjuvancy, sample collection, patient characteristics, definition of response, types of platform used and data analysis. Furthermore, recent investigations suggest the importance not only of the tumor tissue itself but also of the immune system's dysregulation assessed in the peripheral blood of patients with LARC [18]. The lack of large-scale studies and the difficulty to understand and select which data would be informative and useful for a reliable clinical application offer further discussion and variability among the studies [19]. However, considering the utility of gene expression profiling in other tumors, like breast cancer, expression profiling of LARC could be crucial to improve the management of these patients.

The results reported here show the expression patterns of response to CRT in LARC patients. Many of the genes were related to the *Cellular Growth and Proliferation* IPA category, and were over-expressed in patients who responded to treatment. They included a broad range of genes involved in cell-cycle control, DNA synthesis, and c-Myc network such as Gng4, Mapk9, Mcm3, c-Myc, Pola1, Polr2k, and Rrm1, suggesting that LARC cells in CTR-(?) responders present a higher proliferative rate compared to non-responders. Although this hypothesis will need to be confirmed by direct analysis of cell cycle in LARC tumor samples, our results are in agreement with previous studies showing an increased proliferative capacity of tumor cells in patients that respond to treatment [20]. We demonstrated that high Gng4, c-Myc, Pola1, and Rrm1 mRNA expression levels were a significant prognostic factor for response to treatment in LARC patients (p<0.05). Using this gene set, we were able to establish a new model to predict response to radiotherapy in rectal cancer with a sensitivity of 60% and a high specificity of 100%. These findings indicate that up-regulation of these genes could represent an independent predictor of response to treatment in LARC patients. It is necessary to further identify the specific mechanisms involved in this process to further understand the response to treatment of LARC.

Understanding of the function of c-Myc could increase our understanding of the biology of the responder LARC patients but also may provide a novel therapeutic molecular target for clinical practice. However, the prognostic value for the over-expression of c-Myc mRNA has not been analyzed in rectal tumors, and it should be noted that our cohort of patients is small, and that these results will need to be validated in additional patient cohorts and across multiple institutions.

Deregulation and over-expression of c-Myc, in addition to having proliferative effects, is frequently associated with an apoptosis-prone phenotype [21]. Thus opening the possibility of therapeutic intervention due to rapidly proliferating cells are generally more sensitive to chemotherapy. However, the relationship between c-Myc expression and its apoptosis-promoting effects of more clinically relevant chemotherapeutic agents on rectal cancer cells has not been investigated. This could be important since amplification of c-Myc was identified in primary colon tumor patients with increased disease-free (tumor clearance?) and overall survival after 5-fluorouracil (5-FU) based adjuvant therapy, and amplification of c-Myc was shown to result in a further up-regulation of c-Myc “*in vitro*” [22]. In contrast, c-Myc expression was associated with reduced cancer specific survival in rectal cancer patients [23]. We suggest that up-regulation of c-Myc in rectal cancer cells results in a markedly increased sensitivity to apoptosis induced by fluoropyrimidine capecitabine which is enzymatically converted to 5-FU in the tumor, where it inhibits DNA synthesis and slows growth of tumor tissue. Since 5-FU is an S-phase specific drug, and only active during certain cell cycles, those LARC patients, showed higher expression levels of *Cellular Growth and Proliferation* signature, and c-Myc, could respond better to the treatment

Physiologically, c-Myc DNA, mRNA and protein levels are tightly regulated. The high levels of Myc mRNA in LARC could be attributed to gain of c-Myc DNA or aberrant transcriptional activation. However, when we examined rectal tumors before CRT, we found no evidence of gene amplification, according to (which is in agreement with) previous colorectal studies [24]. While higher c-Myc mRNA expression levels correlated with response to treatment, a non significant correlation was found in our study between c-Myc mRNA overexpression in the tumor and c-Myc gene amplification, suggesting that overexpression of c-Myc also can occur via mechanisms independent of gene amplification [25], such as chromosomal translocations [26], point mutations in the coding sequence of the promoter region [27]–[30], or activation/deactivation of trans-activating factors. Finally, we studied the relationship between c-Myc mRNA and protein expression levels. Previous studies have recognized the diagnostic significance of immunohistochemical analysis for c-Myc in human rectal cancers [31] although the limited amounts of pre-treatment biopsy material impede the analysis of c-Myc at the protein level. In interpreting our gene expression data, we generally assume that protein levels in these tissue samples reflect the expression of their corresponding mRNAs. However, both responder and non-responder tumor patients overexpressed c-Myc protein independently of their c-Myc RNA expression levels. Biological reasons for this poor correlation between c-Myc mRNA and protein levels could include post-translational modifications, as well as the protein's half-live [32]. We acknowledge that this needs not to be the case, as post-transcriptional, translational and protein degradation controls probably have a significant influence in LARC. Novel observations from this study were the distinct expression of c-Myc in adenocarcinoma and its adjacent normal tissue samples, and the range of c-Myc protein expression in LARC.

### Conclusion

We demonstrated that high Gng4, c-Myc, Pola1, and Rrm1 mRNA expression levels was a significant prognostic factor for response to treatment in LARC patients. Using this gene set, we were able to establish a new model to predict response to radiotherapy in rectal cancer with a sensitivity of 60% and a high specificity of 100%. Moreover, the information obtained from this study supports the hypothesis that elevated expression of c-Myc mRNA is an important marker of response to CRT in LARC as an essential component of the neoplastic phenotype in rectal tumors.

## Supporting Information

Figure S1 **Rectal tumor samples were clustered according to the expression of 257 genes differentially expressed (p<0.05) between responders and non non-responders tumors.** Rectal cancer samples are across the horizontal axis, with 1 sample expression pattern shown in each column. Tumors with response to treatment appear are colored in grey color while tumors non non-responders tumors are in black color. Gene expression values range from red (over-regulation) to blue (over-expression). Red color represents over-regulation in the gene expression and blue color means over-expression.
(DOC)

Figure S2 **Box plots representing expression values of genes Gng4, c-Myc, Pola1 and Rrm1 genes by quantitative real-time RT-PCR in both groups of rectal cancer patients defined by their response to treatment: responder (R), and non-responder (NR).** Boxes represent the quartiles, median is represented by a black line within the box, and circles (°) show atypical values (1.5–3 times the length of the box). Asterisk (*) shows extreme values (more than three times the box). Significant differences in the expression among responder and non-responder patients were found.
(DOCX)

Table S1 **Specific Taqman primers and probes used in quantitative RT-PCR assays of over-expressed genes in tumor samples from responder rectal cancer patients before treatment.**
(DOCX)

Table S2 **257 genes differentially expressed between responder and non-Responder to treatment LARC patients.**
(DOCX)

Table S3 **Area under curve (AUC) value and 95% confidence interval (CI) were calculated to determine the specificity and sensitivity of response to treatment prediction.**
(DOCX)
