# Supplementary figures and images for: Expression Profiling of Rectal Tumors Defines Response to Neoadjuvant Treatment Related Genes
Source: PLoS One. 2014 Nov 7;9(11):e112189. doi: 10.1371/journal.pone.0112189 (PMC4224421; doi:10.1371/journal.pone.0112189)

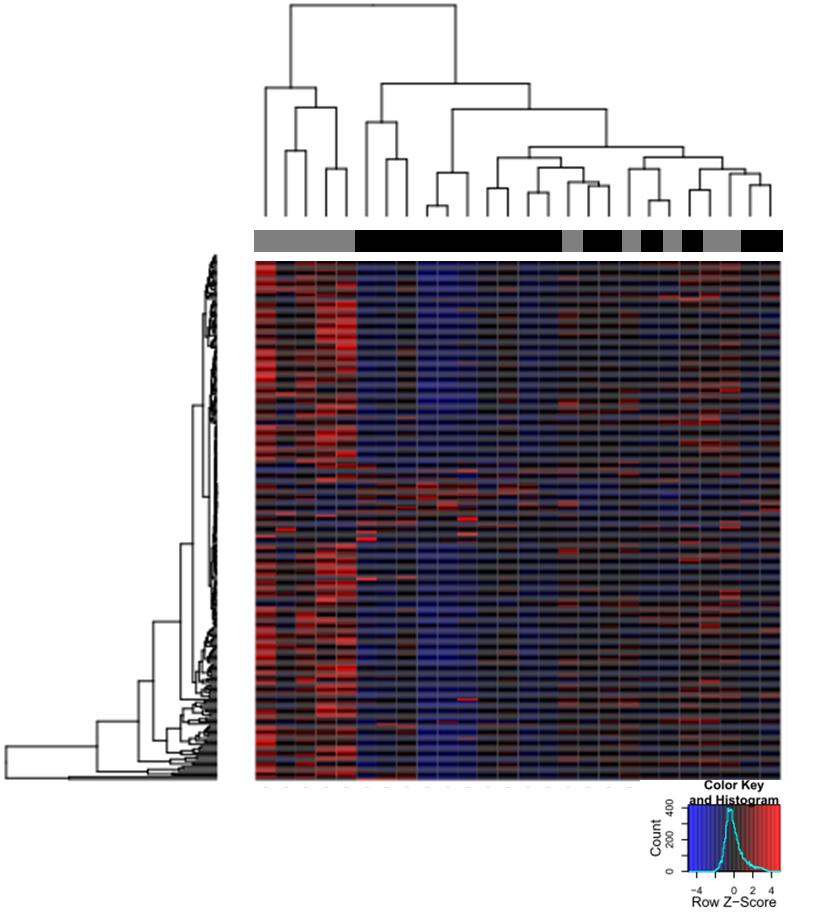

Supplement: Figure S1 — Rectal tumor samples were clustered according to the expression of 257 genes differentially expressed (p<0.05) between responders and non non-responders tumors. Rectal cancer samples are across the horizontal axis, with 1 sample expression pattern shown in each column. Tumors with response to treatment appear are colored in grey color while tumors non non-responders tumors are in black color. Gene expression values range from red (over-regulation) to blue (over-expression). Red color represents over-regulation in the gene expression and blue color means over-expression. (DOC) [file pone.0112189.s001.doc]

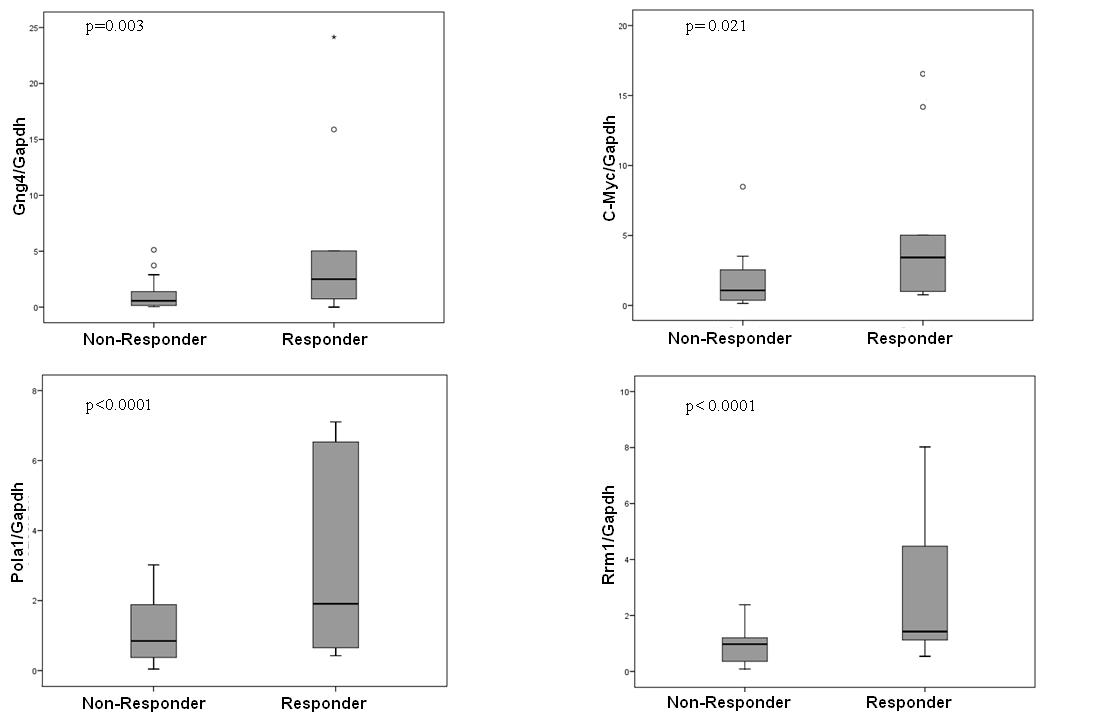

Supplement: Figure S2 — Box plots representing expression values of genes Gng4, c-Myc, Pola1 and Rrm1 genes by quantitative real-time RT-PCR in both groups of rectal cancer patients defined by their response to treatment: responder (R), and non-responder (NR). Boxes represent the quartiles, median is represented by a black line within the box, and circles (°) show atypical values (1.5–3 times the length of the box). Asterisk (*) shows extreme values (more than three times the box). Significant differences in the expression among responder and non-responder patients were found. (DOCX) [file pone.0112189.s002.docx]
