# Supplementary material for: Expression Profiling of Rectal Tumors Defines Response to Neoadjuvant Treatment Related Genes
Source: PLoS One. 2014 Nov 7;9(11):e112189. doi: 10.1371/journal.pone.0112189 (PMC4224421; doi:10.1371/journal.pone.0112189)
Supplement: Table S1 — Specific Taqman primers and probes used in quantitative RT-PCR assays of over-expressed genes in tumor samples from responder rectal cancer patients before treatment. (DOCX) [file pone.0112189.s003.docx]

| **Gene Name** | **Description** | **Type** | **Sequence** |
| --- | --- | --- | --- |
| **GNG4** | guanine nucleotide binding protein (g protein), gamma 4 | Probe | 5´-GCGGACCTCCTGGCCTA-3´ |
|  |  | Primer Forward | 5´-AGATGGAAGCCTGTATGGAC-3´ |
|  |  | Primer Reverse | 5´-ACGTGAGCTTCACAGTAG-3´ |
| **c-MYC** | v-myc myelocytomatosis viral oncogene homolog (avian) | Probe | 5´-AGGCTCCTGGCAAAAGGTC-3´ |
|  |  | Primer Forward | 5´-AGCAGCGACTCTGAGGA-3´ |
|  |  | Primer Reverse | 5´-AAGGTGATCCAGACTCTGAC-3´ |
| **MMP12** | matrix metalloproteinase 12 (macrophage elastase) | Probe | 5´-ACATACGTGGCATTCAGTCC-3´ |
|  |  | Primer Forward | 5´-TCCAAAGGCCGTAATGTTC-3´ |
|  |  | Primer Reverse | 5´-TTGGGTCTCCATACAGG-3´ |
| **ECT2** | epithelial cell transforming sequence 2 oncogene | Probe | 5´-AAAATTCAGGGTTGCTG-3´ |
|  |  | Primer Forward | 5´-GGTGACATTGGTCCATCACAT-3´ |
|  |  | Primer Reverse | 5´-TGGAGTACCTAGACTCACAG-3´ |
| **CRI2** | crebbp/ep300 inhibitor 2 | Probe | 5´-GATAGCTCTGACTGCCTCTG-3´ |
|  |  | Primer Forward | 5´-CAGCGTTTGATGCCGAAT-3´ |
|  |  | Primer Reverse | 5´-GAGGGTTGATAACTTCAG-3´ |
| **NAT5** | n-acetyltransferase 5 (ard1 homolog, s cerevisiae), transcript variant 2 | Probe | 5´-CAGTTCTGTGCCTGAGA-3´ |
|  |  | Primer Forward | 5´-CACTGTATGTATGCTAGGGAAA-3´ |
|  |  | Primer Reverse | 5´-ATATGCAGCAGTGGTTC-3´ |
| **CHMP4B** | chromatin modifying protein 4b | Probe | 5´-GTTTGACGAGGATGAGC-3´ |
|  |  | Primer Forward | 5´-ACCTGTAGGGTTTGGAGAAG-3´ |
|  |  | Primer Reverse | 5´-TCTAATTCCGCCATGAG-3´ |
| **FAM33A** | family with sequence similarity 33, member a | Probe | 5´-CGAGATGTTGAGTGACAGC-3´ |
|  |  | Primer Forward | 5´-TCATGCAGAGGGGCGTTAC-3´ |
|  |  | Primer Reverse | 5´-AGGGCCTCCATTGGACAG-3´ |
| **TOP1MT** | topoisomerase (dna) i, mitochondrial, nuclear gene encoding mitochondrial protein | Probe | 5´-ATCAGCATTGCCTGGTGC-3´ |
|  |  | Primer Forward | 5´-AGCTCAACTACCTGGAC-3´ |
|  |  | Primer Reverse | 5´-CCCTGAACCGCTTGCAC-3´ |
| **SRFBP1** | serum response factor binding protein 1 | Probe | 5´-GAGTCATCAAAGAATGC-3´ |
|  |  | Primer Forward | 5´-GATGTGCTAAAAGCTGCTGTA-3´ |
|  |  | Primer Reverse | 5´-GAATGATTGTCCTCTGAAGC-3´ |
| **RRM1** | ribonucleotide reductase m1 polypeptide | Probe | 5´-TCTATTCAGAGCATACC-3´ |
|  |  | Primer Forward | 5´-TTACCGAGCGGGGCCTAT-3´ |
|  |  | Primer Reverse | 5´-CAGGTCATCAGGAATTTCTGG-3´ |
| **CD81** | cd81 antigen (target of antiproliferative antibody 1 | Probe | 5´-GTGAAGACCTTCCACGAG-3´ |
|  |  | Primer Forward | 5´-TGTGAAGCAGTTCTATGACCAG-3´ |
|  |  | Primer Reverse | 5´-ACAGCAGTCAAGCGTCT-3´ |
| **MAPK9** | mitogen-activated protein kinase 9, transcript variant 2 | Probe | 5´-ACTCATGCAAAGAGAGC-3´ |
|  |  | Primer Forward | 5´-CCCAAGGGATTGTTTGTGCT-3´ |
|  |  | Primer Reverse | 5´-GGACAAGTTCACGATAAGCTC-3´ |
| **STMN1** | stathmin 1/oncoprotein 18, transcript variant 1 | Probe | 5´-TATCCAGGTGAAAGAACT-3´ |
|  |  | Primer Forward | 5´-AGAATACACTGCCTGTCGCTTG-3´ |
|  |  | Primer Reverse | 5´-AGGACGCTTCTCCAGTT-3´ |
| **ID1** | inhibitor of dna binding 1, dominant negative helix-loop-helix protein , transcript variant 2 | Probe | 5´-TCTACGACATGAACGGCTGT-3´ |
|  |  | Primer Forward | 5´-CTGTCTGTCTGAGCAGAG-3´ |
|  |  | Primer Reverse | 5´-CCTTGAGGCGTGAGTAAC-3´ |
| **DPM1** | dolichyl-phosphate mannosyltransferase polypeptide 1, catalytic subunit | Probe | 5´-TACTATTGGCGAGGTTCC-3´ |
|  |  | Primer Forward | 5´-AAGGCTACGTCTTCCAGATG-3´ |
|  |  | Primer Reverse | 5´-CACGATCCACAAATGATATTGG-3´ |
| **STMN2** | stathmin 1/oncoprotein 18 (stmn1), transcript variant 2 | Probe | 5´-GGAAAGAAGAAAGTCTCAGG-3´ |
|  |  | Primer Forward | 5´-GCTGATCTTGAAGCCACCAT-3´ |
|  |  | Primer Reverse | 5´-TTCAGCACCTGGGCCTCCTG-3´ |
| **P53CSV** | p53-inducible cell-survival factor | Probe | 5´-GGAGAAAGAGATTCCTA-3´ |
|  |  | Primer Forward | 5´-CCAGCAGTGTGTTCAGAAAG-3´ |
|  |  | Primer Reverse | 5´-GAACTCCAGTCCTTCAATAGG-3´ |
| **POLA** | yw15b01r1 soares_placenta_8to9weeks_2nbhp8to9w cdna clone image:252265 5' similar to gb:x60489 elongation factor 1-beta (human) | Probe | 5´-ATTTGCTGGCGATGATGT-3´ |
|  |  | Primer Forward | 5´-GAGCCTCCATTAACTCCTGT-3´ |
|  |  | Primer Reverse | 5´-TGTACTCTCTCGACCTGTA-3´ |
| **ABCB7** | atp-binding cassette, sub-family b (mdr/tap), member 7, nuclear gene encoding mitochondrial protein | Probe | 5´-CAGCAGTTCTGATTGGC-3´ |
|  |  | Primer Forward | 5´-TGTAGACAGCCTCAACCAGATG-3´ |
|  |  | Primer Reverse | 5´-GCTCTTGATACACCATAGCC-3´ |
| **TP53** | tumor protein p53 | Probe | 5´-CCCTATGAGCCGCCTGA-3´ |
|  |  | Primer Forward | 5´-CTCAGCATCTTATCCGAGTGGAAG-3´ |
|  |  | Primer Reverse | 5´-TGGTACAGTCAGAGCCAACCTCA-3´ |
| **CDKN1A** | cyclin-dependent kinase inhibitor 1a | Probe | 5´-GAACTTCGACTTTGTCACCG-3´ |
|  |  | Primer Forward | 5´-GCGACTGTGATGCGCTAA-3´ |
|  |  | Primer Reverse | 5´-CCTCCAGTGGTGTCTCGGT-3´ |
