# Supplementary material for: Expression Profiling of Rectal Tumors Defines Response to Neoadjuvant Treatment Related Genes
Source: PLoS One. 2014 Nov 7;9(11):e112189. doi: 10.1371/journal.pone.0112189 (PMC4224421; doi:10.1371/journal.pone.0112189)
Supplement: Table S2 — 257 genes differentially expressed between responder and non-Responder to treatment LARC patients. (DOCX) [file pone.0112189.s004.docx]

| **Gene Name** | **CodeLink ID** | **Description** | **Fold change** |
| --- | --- | --- | --- |
| GNG4 | NM_004485.2 | guanine nucleotide binding protein (g protein), gamma 4 (gng4) | 3,00 |
| c-MYC | NM_002467.3 | v-myc myelocytomatosis viral oncogene homolog (avian) (myc), mrna | 2,98 |
| MMP12 | NM_002426.1 | matrix metalloproteinase 12 (macrophage elastase) (mmp12) | 2,30 |
| HSPCP1 | BQ431029.1 | agencourt_7766536 nih_mgc_68 cdna clone image:6022150 5' | 2,22 |
| STARD7 | NM_139267.1 | start domain containing 7 (stard7), transcript variant 2 | 2,01 |
| GEMIN5 | NM_015465.1 | gem (nuclear organelle) associated protein 5 (gemin5) | 1,96 |
| ECT2 | NM_018098.4 | epithelial cell transforming sequence 2 oncogene (ect2) | 1,78 |
| CRI2 | NM_153232.3 | crebbp/ep300 inhibitor 2 (cri2) | 1,77 |
| AK092401.1 | AK092401.1 | cdna flj35082 fis, clone place6005351 | 1,76 |
| NAT5 | NM_181527.1 | n-acetyltransferase 5 (ard1 homolog, s cerevisiae) (nat5), transcript variant 2 | 1,74 |
| CHMP4B | NM_176812.3 | chromatin modifying protein 4b (chmp4b) | 1,72 |
| BF057809.1 | BF057809.1 | 7k54b08x1 nci_cgap_gc6 cdna clone image:3479007 3' | 1,72 |
| FAM33A | NM_182620.1 | family with sequence similarity 33, member a (fam33a) | 1,70 |
| C1orf33 | W77904.1 | zd70f05r1 soares_fetal_heart_nbhh19w cdna clone image:346017 5' similar to contains alu repetitive element;contains element ptr5 repetitive element | 1,69 |
| LOC151475 | AK055016.1 | cdna flj30454 fis, clone brace2009311 | 1,66 |
| TOP1MT | NM_052963.1 | topoisomerase (dna) i, mitochondrial (top1mt), nuclear gene encoding mitochondrial protein | 1,65 |
| CR595760.1 | CR595760.1 | full-length cdna clone cs0dh004yf14 of t cells (jurkat cell line) of homo sapiens (human) | 1,63 |
| SRFBP1 | NM_152546.1 | serum response factor binding protein 1 (srfbp1) | 1,60 |
| MGC13170 | NM_199250.1 | multidrug resistance-related protein (mgc13170) | 1,60 |
| RRM1 | NM_001033.2 | ribonucleotide reductase m1 polypeptide (rrm1) | 1,59 |
| CD81 | NM_004356.3 | cd81 antigen (target of antiproliferative antibody 1) (cd81) | 1,58 |
| TOM1L1 | NM_005486.1 | target of myb1-like 1 (chicken) (tom1l1) | 1,56 |
| RFWD3 | NM_018124.3 | ring finger and wd repeat domain 3 (rfwd3), mrna | 1,54 |
| MAPK9 | NM_139068.1 | mitogen-activated protein kinase 9 (mapk9), transcript variant 2 | 1,53 |
| GLCE | BQ889128.1 | agencourt_8120808 lupski_dorsal_root_ganglion cdna clone image:6178590 5' | 1,53 |
| CXorf39 | BX116609.1 | soares infant brain 1nib cdna clone imagp998b12166 | 1,52 |
| COX11 | NM_004375.2 | cox11 homolog, cytochrome c oxidase assembly protein (yeast) (cox11), nuclear gene encoding mitochondrial protein | 1,52 |
| CASC5 | AA662240.1 | nu89c01s1 nci_cgap_alv1 cdna clone image:1217856 | 1,51 |
| FAM98A | NM_015475.3 | dkfzp564f0522 protein (dkfzp564f0522) | 1,50 |
| NOLA1 | NM_018983.2 | nucleolar protein family a, member 1 (h/aca small nucleolar rnps) (nola1), transcript variant 1 | 1,50 |
| TPD52L1 | NM_001003395.1 | tumor protein d52-like 1 (tpd52l1), transcript variant 2 | 1,50 |
| STMN1 | NM_203399.1 | stathmin 1/oncoprotein 18 (stmn1), transcript variant 2 | 1,50 |
| SFRS6 | U30828.1 | splicing factor srp55-2 (srp55) mrna | 1,49 |
| AGMAT | NM_024758.3 | agmatine ureohydrolase (agmatinase) (agmat) | 1,49 |
| ID1 | NM_181353.1 | inhibitor of dna binding 1, dominant negative helix-loop-helix protein (id1), transcript variant 2 | 1,45 |
| OIP5 | NM_007280.1 | opa interacting protein 5 (oip5) | 1,45 |
| BU736752.1 | BU736752.1 | ui-e-ci1-afo-b-13-0-uis2 ui-e-ci1 cdna clone ui-e-ci1-afo-b-13-0-ui 3' | 1,43 |
| LSM5 | NM_012322.1 | lsm5 homolog, u6 small nuclear rna associated (s cerevisiae) (lsm5) | 1,43 |
| UBA2 | NM_005499.2 | sumo-1 activating enzyme subunit 2 (uba2) | 1,41 |
| CGI-115 | NM_016052.2 | cgi-115 protein (cgi-115) | 1,40 |
| GLT28D1 | NM_018466.2 | glycosyltransferase 28 domain containing 1 (glt28d1) | 1,40 |
| GLOXD1 | NM_032756.2 | hypothetical protein mgc15668 (mgc15668) | 1,39 |
| C17orf66 | BG777291.1 | 602664527f1 nih_mgc_59 cdna clone image:4809606 5' | 1,37 |
| WDSOF1 | NM_015420.4 | wd repeats and sof1 domain containing (wdsof1) | 1,36 |
| BRCTD1 | CR936768.1 | mrna; cdna dkfzp686e22228 (from clone dkfzp686e22228) | 1,36 |
| N55958.1 | N55958.1 | j4702f human fetal heart, lambda zap express cdna clone j4702 5' | 1,35 |
| DPM1 | NM_003859.1 | dolichyl-phosphate mannosyltransferase polypeptide 1, catalytic subunit (dpm1) | 1,35 |
| NLE1 | BE906938.1 | 601500740f1 nih_mgc_70 cdna clone image:3902817 5' | 1,35 |
| WRB | NM_004627.2 | tryptophan rich basic protein (wrb) | 1,34 |
| ATRN | NM_139322.1 | attractin (atrn), transcript variant 2 | 1,33 |
| AW135639.1 | AW135639.1 | ui-h-bi1-acc-f-01-0-uis1 nci_cgap_sub3 cdna clone image:2713872 3' | 1,33 |
| GTF2F2 | NM_004128.1 | general transcription factor iif, polypeptide 2 (30kd subunit) (gtf2f2) | 1,32 |
| BU634151.1 | BU634151.1 | ui-h-fl1-bgw-h-18-0-uis1 nci_cgap_fl1 cdna clone ui-h-fl1-bgw-h-18-0-ui 3' | 1,32 |
| MCM3 | NM_002388.3 | mcm3 minichromosome maintenance deficient 3 (s cerevisiae) (mcm3) | 1,31 |
| RIF1 | NM_018151.3 | rap1 interacting factor homolog (yeast) (rif1) | 1,31 |
| FTSJ2 | NM_177442.1 | ftsj homolog 2 (e coli) (ftsj2), transcript variant 2 | 1,30 |
| NUP160 | NM_015231.1 | nucleoporin 160kda (nup160) | 1,30 |
| RKHD1 | NM_203304.1 | ring finger and kh domain containing 1 (rkhd1) | 1,30 |
| TM7SF3 | BM838078.1 | k-est0114256 s9snu601 cdna clone s9snu601-74-h04 5' | 1,30 |
| UPF3B | NM_080632.1 | upf3 regulator of nonsense transcripts homolog b (yeast) (upf3b), transcript variant 1 | 1,30 |
| AK095831.1 | AK095831.1 | cdna flj38512 fis, clone hchon2000503 | 1,29 |
| LOC158381 | BC035229.1 | hypothetical protein loc158381, mrna (cdna clone image:4824906), with apparent retained intron | 1,29 |
| C4orf14 | NM_032313.2 | chromosome 4 open reading frame 14 (c4orf14) | 1,29 |
| BQ277155.1 | BQ277155.1 | agencourt_6824167 nih_mgc_127 cdna clone image:5810333 5' | 1,28 |
| COPS4 | NM_016129.2 | cop9 constitutive photomorphogenic homolog subunit 4 (arabidopsis) (cops4) | 1,27 |
| C6orf166 | NM_018064.2 | chromosome 6 open reading frame 166 (c6orf166) | 1,27 |
| C14orf109 | BU739864.1 | ui-e-ej0-ahu-h-15-0-uis1 ui-e-ej0 cdna clone ui-e-ej0-ahu-h-15-0-ui 3' | 1,26 |
| TIMM13 | NM_012458.2 | translocase of inner mitochondrial membrane 13 homolog (yeast) (timm13), nuclear gene encoding mitochondrial protein | 1,26 |
| CR621142.1 | CR621142.1 | full-length cdna clone cs0dn003ye14 of adult brain of homo sapiens (human) | 1,25 |
| SV2C | T08682.1 | est06574 infant brain, bento soares cdna clone hibbi28 5' end similar to rap1b ras-related protein | 1,25 |
| ZNF137 | NM_003438.1 | zinc finger protein 137 (clone phz-30) (znf137) | 1,24 |
| ROD1 | CR749471.1 | mrna; cdna dkfzp781i1117 (from clone dkfzp781i1117) | 1,24 |
| BF574216.1 | BF574216.1 | 602131380f1 nih_mgc_81 cdna clone image:4271082 5' | 1,23 |
| STX3A | F31730.1 | hspd23129 hm3 cdna clone s4000125d09 | 1,22 |
| PEX13 | NM_002618.2 | peroxisome biogenesis factor 13 (pex13) | 1,22 |
| SDAD1 | NM_018115.1 | sda1 domain containing 1 (sdad1) | 1,22 |
| GMNN | NM_015895.3 | geminin, dna replication inhibitor (gmnn) | 1,21 |
| NME2 | NM_001018139.1 | non-metastatic cells 2, protein (nm23b) expressed in (nme2), transcript variant 5, mrna | 1,21 |
| BM553569.1 | BM553569.1 | agencourt_6584538 nih_mgc_41 cdna clone image:5472722 5' | 1,21 |
| PMCH | BE538546.1 | 601065310f1 nih_mgc_10 cdna clone image:3451706 5' | 1,20 |
| NPM3 | NM_006993.1 | nucleophosmin/nucleoplasmin, 3 (npm3) | 1,20 |
| NDUFB5 | NM_002492.2 | nadh dehydrogenase (ubiquinone) 1 beta subcomplex, 5, 16kda (ndufb5), nuclear gene encoding mitochondrial protein | 1,20 |
| SMC1L1 | D80000.2 | kiaa0178 mrna | 1,20 |
| ZW10 | NM_004724.2 | zw10 homolog, centromere/kinetochore protein (drosophila) (zw10) | 1,20 |
| CA455253.1 | CA455253.1 | agencourt_10640955 nih_mgc_126 cdna clone image:6723568 5' | 1,20 |
| RP11-484I6.3 | NM_138779.2 | hypothetical protein bc015148 (loc93081) | 1,20 |
| TCFL5 | NM_006602.2 | transcription factor-like 5 (basic helix-loop-helix) (tcfl5) | 1,19 |
| FLJ20272 | NM_017735.3 | hypothetical protein flj20272 (flj20272) | 1,19 |
| DERL1 | NM_024295.3 | der1-like domain family, member 1 (derl1) | 1,18 |
| STMN2 | AK092187.1 | cdna flj34868 fis, clone nt2ne2014525, highly similar to scg10 protein | 1,18 |
| BQ054490.1 | BQ054490.1 | agencourt_6771241 nih_mgc_99 cdna clone image:5803764 5' | 1,18 |
| DDX28 | NM_018380.2 | dead (asp-glu-ala-asp) box polypeptide 28 (ddx28), nuclear gene encoding mitochondrial protein | 1,17 |
| MYOHD1 | NM_025109.3 | myosin head domain containing 1 (myohd1) | 1,17 |
| RNF113B | NM_178861.3 | ring finger protein 113b (rnf113b) | 1,17 |
| MGC2747 | NM_024104.2 | hypothetical protein mgc2747 (mgc2747) | 1,17 |
| BQ073592.1 | BQ073592.1 | agencourt_7046491 nih_mgc_101 cdna clone image:5806429 5' | 1,16 |
| CXCL3 | NM_002090.2 | chemokine (c-x-c motif) ligand 3 (cxcl3) | 1,15 |
| SPAST | NM_199436.1 | spastin (spast), transcript variant 2 | 1,14 |
| KHK | NM_006488.1 | ketohexokinase (fructokinase) (khk), transcript variant b | 1,14 |
| C2orf3 | AB026911.1 | gcf2 fusion protein | 1,13 |
| CNP | AW896213.1 | pm0-nn0046-040400-001-a07 nn0046 homo sapiens cdna | 1,13 |
| SEPN1 | BQ678662.1 | agencourt_8210468 nih_mgc_112 cdna clone image:6259260 5' | 1,12 |
| C2orf33 | NM_020194.4 | chromosome 2 open reading frame 33 (c2orf33) | 1,11 |
| TMEM41A | NM_080652.2 | transmembrane protein 41a (tmem41a) | 1,10 |
| PIGA | NM_020472.1 | phosphatidylinositol glycan, class a (paroxysmal nocturnal hemoglobinuria) (piga), transcript variant 2 | 1,10 |
| LOC51136 | NM_016125.2 | ptd016 protein (loc51136) | 1,10 |
| NCBP2 | NM_007362.2 | nuclear cap binding protein subunit 2, 20kda (ncbp2) | 1,09 |
| MRPL9 | NM_031420.2 | mitochondrial ribosomal protein l9 (mrpl9), nuclear gene encoding mitochondrial protein | 1,09 |
| C15orf23 | CR602848.1 | full-length cdna clone cs0db002yk23 of neuroblastoma cot 10-normalized of homo sapiens (human) | 1,08 |
| C18orf37 | NM_194281.2 | chromosome 18 open reading frame 37 (c18orf37) | 1,08 |
| ZNF35 | NM_003420.2 | zinc finger protein 35 (clone hf10) (znf35) | 1,08 |
| CR616734.1 | CR616734.1 | full-length cdna clone cs0di027yf17 of placenta cot 25-normalized of homo sapiens (human) | 1,08 |
| P53CSV | NM_016399.2 | p53-inducible cell-survival factor (p53csv) | 1,07 |
| FAHD1 | NM_031208.1 | fumarylacetoacetate hydrolase domain containing 1 (fahd1), transcript variant 2 | 1,07 |
| FAM82B | NM_016033.1 | cgi-90 protein (cgi-90) | 1,06 |
| UBE3A | NM_000462.2 | ubiquitin protein ligase e3a (human papilloma virus e6-associated protein, angelman syndrome) (ube3a), transcript variant 2 | 1,06 |
| KRT8 | CB147628.1 | k-est0203659 l14choick0 cdna clone l14choick0-17-a10 5' | 1,06 |
| EIF2A | NM_032025.2 | eukaryotic translation initiation factor (eif) 2a (eif2a) | 1,06 |
| POLA | H87655.1 | yw15b01r1 soares_placenta_8to9weeks_2nbhp8to9w cdna clone image:252265 5' similar to gb:x60489 elongation factor 1-beta (human) | 1,06 |
| C7orf24 | NM_024051.2 | chromosome 7 open reading frame 24 (c7orf24) | 1,06 |
| MRPL11 | NM_170738.1 | mitochondrial ribosomal protein l11 (mrpl11), nuclear gene encoding mitochondrial protein, transcript variant 2 | 1,05 |
| MRPL12 | NM_002949.2 | mitochondrial ribosomal protein l12 (mrpl12), nuclear gene encoding mitochondrial protein | 1,05 |
| PPAP2C | NM_177526.1 | phosphatidic acid phosphatase type 2c (ppap2c), transcript variant 2 | 1,05 |
| SS18L2 | NM_016305.1 | synovial sarcoma translocation gene on chromosome 18-like 2 (ss18l2) | 1,05 |
| MRPS15 | NM_031280.2 | mitochondrial ribosomal protein s15 (mrps15), nuclear gene encoding mitochondrial protein | 1,05 |
| RAD18 | NM_020165.2 | rad18 homolog (s cerevisiae) (rad18) | 1,04 |
| C6orf211 | NM_024573.1 | chromosome 6 open reading frame 211 (c6orf211) | 1,04 |
| COMMD8 | NM_017845.2 | comm domain containing 8 (commd8) | 1,04 |
| KIAA0241 | NM_015060.1 | kiaa0241 protein (kiaa0241) | 1,04 |
| NT5C3 | NM_001002009.1 | 5'-nucleotidase, cytosolic iii (nt5c3), transcript variant 2 | 1,04 |
| TEX10 | NM_017746.1 | testis expressed sequence 10 (tex10) | 1,03 |
| SEPT10 | BE618882.1 | 601462916t1 nih_mgc_67 cdna clone image:3866354 3' | 1,03 |
| RPUSD4 | NM_032795.1 | rna pseudouridylate synthase domain containing 4 (rpusd4) | 1,02 |
| RPL5 | NM_000969.3 | ribosomal protein l5 (rpl5), mrna | 1,02 |
| MGC11257 | BG337819.1 | 602435722f1 nih_mgc_46 cdna clone image:4553620 5' | 1,02 |
| EXOSC2 | NM_014285.4 | exosome component 2 (exosc2) | 1,02 |
| PRKAR1A | M33336.1 | camp-dependent protein kinase type i-alpha subunit (prkar1a) mrna | 1,02 |
| DRB1 | NM_152945.1 | developmentally regulated rna-binding protein 1 (drb1) | 1,01 |
| DARS | NM_001349.2 | aspartyl-trna synthetase (dars) | 1,01 |
| PHF22 | NM_020395.2 | phd finger protein 22 (phf22) | 1,00 |
| SYNCRIP | AK222776.1 | synaptotagmin binding, cytoplasmic rna interacting protein variant, clone: hep01044 | 1,00 |
| T93589.1 | T93589.1 | ye17g06s1 stratagene lung (#937210) cdna clone image:118042 3' similar to gb:k00558 tubulin alpha-1 chain (human) | 1,00 |
| ABCB7 | NM_004299.3 | atp-binding cassette, sub-family b (mdr/tap), member 7 (abcb7), nuclear gene encoding mitochondrial protein | 0,98 |
| MRPL45 | NM_032351.3 | mitochondrial ribosomal protein l45 (mrpl45), nuclear gene encoding mitochondrial protein | 0,98 |
| HSPC196 | NM_016464.2 | hypothetical protein hspc196 (hspc196) | 0,98 |
| ARD1A | NM_003491.2 | ard1 homolog a, n-acetyltransferase (s cerevisiae) (ard1a) | 0,97 |
| TncRNA | BE177702.1 | rc1-ht0598-140300-021-c09 ht0598 homo sapiens cdna | 0,97 |
| NBLA00058 | NM_019048.1 | hcv ns3-transactivated protein 1 (ns3tp1) | 0,97 |
| NUDCD2 | NM_145266.4 | nudc domain containing 2 (nudcd2) | 0,97 |
| PPHLN1 | NM_201439.1 | periphilin 1 (pphln1), transcript variant 3 | 0,97 |
| SLC12A2 | NM_001046.2 | solute carrier family 12 (sodium/potassium/chloride transporters), member 2 (slc12a2) | 0,97 |
| C6orf153 | NM_033112.2 | chromosome 6 open reading frame 153 (c6orf153) | 0,96 |
| C1orf109 | NM_017850.1 | chromosome 1 open reading frame 109 (c1orf109) | 0,96 |
| AI821277.1 | AI821277.1 | ac26f02x5 stratagene ovary (#937217) cdna clone image:857595 3' | 0,96 |
| EBPL | BC092471.1 | emopamil binding protein-like, mrna (cdna clone mgc:104589 image:4796715) | 0,96 |
| DSCR2 | NM_003720.2 | down syndrome critical region gene 2 (dscr2), transcript variant 1 | 0,95 |
| BF692405.1 | BF692405.1 | 602247916f1 nih_mgc_62 cdna clone image:4333299 5' | 0,95 |
| INCYTE UNIQUE | INCYTE UNIQUE | incyte unique | 0,94 |
| IQWD1 | H87917.1 | yw16h05r1 soares_placenta_8to9weeks_2nbhp8to9w cdna clone image:252441 5' similar to gb:d00723 glycine cleavage system h protein precursor (human);contains tar1 repetitive element | 0,94 |
| RTCD1 | NM_003729.1 | rna terminal phosphate cyclase domain 1 (rtcd1) | 0,94 |
| CB049479.1 | CB049479.1 | nisc_gj11b07y1 nci_cgap_pr28 cdna clone image:3271260 5' | 0,94 |
| BQ024427.1 | BQ024427.1 | ui-1-bb1p-auq-g-04-0-uis1 nci_cgap_pl6 cdna clone ui-1-bb1p-auq-g-04-0-ui 3' | 0,93 |
| MDH1 | NM_005917.2 | malate dehydrogenase 1, nad (soluble) (mdh1) | 0,92 |
| C10orf85 | NM_001012711.1 | chromosome 10 open reading frame 85 (c10orf85) | 0,91 |
| EML4 | NM_019063.2 | echinoderm microtubule associated protein like 4 (eml4) | 0,91 |
| BE858761.1 | BE858761.1 | 7g04g08x1 nci_cgap_brn23 cdna clone image:3305534 3' similar to contains alu repetitive element;contains element mer32 repetitive element | 0,90 |
| NDUFC1 | NM_002494.2 | nadh dehydrogenase (ubiquinone) 1, subcomplex unknown, 1, 6kda (ndufc1) | 0,90 |
| COL22A1 | AA977081.1 | oq23c07s1 nci_cgap_gc4 cdna clone image:1587180 3' similar to sw:ca24_ascsu p27393 procollagen alpha 2(iv) chain precursor. ;contains mer5.b3 mer5 repetitive element | 0,90 |
| SF3B14 | NM_016047.3 | splicing factor 3b, 14 kda subunit (sf3b14) | 0,90 |
| POLR2K | NM_005034.3 | polymerase (rna) ii (dna directed) polypeptide k, 70kda (polr2k) | 0,90 |
| CYP27B1 | NM_000785.2 | cytochrome p450, family 27, subfamily b, polypeptide 1 (cyp27b1), nuclear gene encoding mitochondrial protein | 0,90 |
| HNRPA3P1 | S63912.1 | d10s102=fbrnp [human, fetal brain, mrna, 3043 nt] | 0,89 |
| CFLAR | NM_003879.3 | casp8 and fadd-like apoptosis regulator (cflar) | 0,89 |
| RUVBL2 | NM_006666.1 | ruvb-like 2 (e coli) (ruvbl2) | 0,88 |
| SLA2 | AA195893.1 | zp97e03r1 stratagene muscle 937209 cdna clone image:628156 5' | 0,87 |
| C7orf9 | NM_022150.2 | chromosome 7 open reading frame 9 (c7orf9) | 0,87 |
| LOC134145 | NM_199133.1 | hypothetical protein loc134145 (loc134145) | 0,87 |
| SAAL1 | NM_138421.1 | hypothetical protein bc012010 (loc113174) | 0,86 |
| MRPL30 | NM_145212.1 | mitochondrial ribosomal protein l30 (mrpl30), nuclear gene encoding mitochondrial protein, transcript variant 1 | 0,86 |
| T91583.1 | T91583.1 | ye21g03s1 stratagene lung (#937210) cdna clone image:118420 3' similar to gb:m26326 keratin, type i cytoskeletal 18 (human); | 0,86 |
| STAMBPL1 | NM_020799.2 | associated molecule with the sh3 domain of stam (amsh) like protein (amsh-lp) | 0,86 |
| SEC23A | NM_006364.2 | sec23 homolog a (s cerevisiae) (sec23a) | 0,85 |
| C20orf9 | NM_016004.2 | chromosome 20 open reading frame 9 (c20orf9) | 0,84 |
| FUCA2 | NM_032020.3 | fucosidase, alpha-l- 2, plasma (fuca2) | 0,82 |
| HIST1H3C | NM_003531.2 | histone 1, h3c (hist1h3c) | 0,82 |
| KIAA1799 | NM_032437.1 | kiaa1799 protein (kiaa1799) | 0,82 |
| MGC12981 | BF918530.1 | cm0-nt0132-121000-611-h05 nt0132 homo sapiens cdna | 0,81 |
| SFRS4 | CR595944.1 | full-length cdna clone cs0dm006yk24 of fetal liver of homo sapiens (human) | 0,81 |
| SNX6 | F27470.1 | hspd15420 hm3 cdna clone s4000071a08 | 0,81 |
| BQ950358.1 | BQ950358.1 | agencourt_8883158 lupski_sciatic_nerve cdna clone image:6200099 5' | 0,80 |
| MRPL1 | NM_020236.2 | mitochondrial ribosomal protein l1 (mrpl1), nuclear gene encoding mitochondrial protein | 0,80 |
| UTP11L | NM_016037.2 | utp11-like, u3 small nucleolar ribonucleoprotein, (yeast) (utp11l) | 0,79 |
| PSMC2 | NM_002803.2 | proteasome (prosome, macropain) 26s subunit, atpase, 2 (psmc2) | 0,79 |
| BM716531.1 | BM716531.1 | ui-e-ej0-ahi-k-13-0-uir2 ui-e-ej0 cdna clone ui-e-ej0-ahi-k-13-0-ui 5' | 0,79 |
| DSCAML1 | BQ187536.1 | ui-e-ej1-ajt-a-05-0-uir1 ui-e-ej1 cdna clone ui-e-ej1-ajt-a-05-0-ui 5' | 0,79 |
| APC | BE047584.1 | tz39a05y1 nci_cgap_brn52 cdna clone image:2290928 5' similar to gb:x12791 19 kd protein of signal recognition particle (human) | 0,77 |
| LOC91137 | NM_138773.1 | hypothetical protein bc017169 (loc91137) | 0,77 |
| STRBP | NM_018387.2 | spermatid perinuclear rna binding protein (strbp) | 0,77 |
| C6orf96 | NM_017909.1 | chromosome 6 open reading frame 96 (c6orf96) | 0,77 |
| YARS2 | NM_015936.1 | tyrosyl-trna synthetase 2 (mitochondrial) (yars2) | 0,76 |
| PRKRIP1 | BU073748.1 | in24a12y1 human fetal pancreas 1b cdna clone image: 5' similar to tr:q13401 q13401 hpmsr3 | 0,76 |
| FKBP3 | NM_002013.2 | fk506 binding protein 3, 25kda (fkbp3) | 0,75 |
| EIF3S12 | NM_013234.1 | eukaryotic translation initiation factor 3, subunit 12 (eif3s12) | 0,75 |
| BQ575336.1 | BQ575336.1 | ui-h-ez1-bbe-l-13-0-uis1 nci_cgap_ch2 cdna clone ui-h-ez1-bbe-l-13-0-ui 3' | 0,75 |
| FUSIP1 | AK054635.1 | cdna flj30073 fis, clone astro2000480 | 0,75 |
| C6orf157 | NM_198920.1 | chromosome 6 open reading frame 157 (c6orf157) | 0,75 |
| MDH2 | NM_005918.2 | malate dehydrogenase 2, nad (mitochondrial) (mdh2) | 0,74 |
| SEMA4A | AK022416.1 | cdna flj12354 fis, clone mamma1002329, weakly similar to mmusculus mrna for semaphorin b | 0,73 |
| SYAP1 | NM_032796.2 | synapse associated protein 1, sap47 homolog (drosophila) (syap1) | 0,73 |
| FAM96B | NM_016062.1 | cgi-128 protein (cgi-128) | 0,72 |
| SCC-112 | NM_015200.1 | scc-112 protein (scc-112) | 0,72 |
| BF690421.1 | BF690421.1 | 602186866t1 nih_mgc_49 cdna clone image:4298825 3' | 0,71 |
| COMMD4 | AK124968.1 | cdna flj42978 fis, clone brtha2004821 | 0,69 |
| SS18L1 | NM_198935.1 | synovial sarcoma translocation gene on chromosome 18-like 1 (ss18l1), transcript variant 1 | 0,69 |
| USP7 | NM_003470.1 | ubiquitin specific protease 7 (herpes virus-associated) (usp7) | 0,69 |
| C11orf24 | NM_022338.2 | chromosome 11 open reading frame 24 (c11orf24) | 0,68 |
| C18orf55 | NM_014177.1 | chromosome 18 open reading frame 55 (c18orf55) | 0,68 |
| RPP30 | NM_006413.2 | ribonuclease p/mrp 30kda subunit (rpp30) | 0,67 |
| C12orf10 | NM_021640.2 | chromosome 12 open reading frame 10 (c12orf10) | 0,67 |
| CBX3 | NM_007276.3 | chromobox homolog 3 (hp1 gamma homolog, drosophila) (cbx3), transcript variant 1 | 0,66 |
| HBS1L | U87791.1 | erfs mrna | 0,66 |
| CUL2 | NM_003591.2 | cullin 2 (cul2) | 0,66 |
| FTH1 | BM913228.1 | agencourt_6613829 nih_mgc_41 cdna clone image:5475270 5' | 0,65 |
| GTF2B | NM_001514.3 | general transcription factor iib (gtf2b) | 0,65 |
| LIG3 | NM_013975.1 | ligase iii, dna, atp-dependent (lig3), transcript variant alpha | 0,65 |
| C21orf66 | BC062992.1 | cdna clone image:5497083, containing frame-shift errors | 0,65 |
| CCDC55 | NM_032141.1 | hypothetical protein dkfzp434k1421 (dkfzp434k1421) | 0,65 |
| RBM15B | W68531.1 | zd37e05s1 soares_fetal_heart_nbhh19w cdna clone image:342848 3' | 0,64 |
| SPIN | AK027055.1 | cdna: flj23402 fis, clone hep18853 | 0,64 |
| PSMA1 | NM_148976.1 | proteasome (prosome, macropain) subunit, alpha type, 1 (psma1), transcript variant 1 | 0,64 |
| DN994689.1 | DN994689.1 | adult whole brain, large insert, pcmv expression library cdna clone tc114241 5' similar to homo sapiens apc11 anaphase promoting complex subunit 11 homolog (yeast) (anapc11), transcript variant 2 | 0,61 |
| RCE1 | AL713740.1 | mrna; cdna dkfzp761d0521 (from clone dkfzp761d0521) | 0,61 |
| C12orf5 | NM_020375.1 | chromosome 12 open reading frame 5 (c12orf5) | 0,60 |
| MRPL51 | NM_016497.2 | mitochondrial ribosomal protein l51 (mrpl51), nuclear gene encoding mitochondrial protein | 0,60 |
| BHLHB9 | AK054934.1 | cdna flj30372 fis, clone brace2007868 | 0,59 |
| SNRPD2 | NM_177542.1 | small nuclear ribonucleoprotein d2 polypeptide 165kda (snrpd2), transcript variant 2 | 0,58 |
| MRPL46 | NM_022163.2 | mitochondrial ribosomal protein l46 (mrpl46), nuclear gene encoding mitochondrial protein | 0,58 |
| CB046341.1 | CB046341.1 | nisc_gf03h02x2 nci_cgap_kid12 cdna clone image:3252722 3' | 0,58 |
| COASY | NM_025233.4 | coenzyme a synthase (coasy) | 0,57 |
| NIF3L1BP1 | NM_025075.1 | ngg1 interacting factor 3 like 1 binding protein 1 (nif3l1bp1) | 0,57 |
| BM726450.1 | BM726450.1 | ui-e-ej0-aii-c-11-0-uir1 ui-e-ej0 cdna clone ui-e-ej0-aii-c-11-0-ui 5' | 0,56 |
| ITGA6 | NM_000210.1 | integrin, alpha 6 (itga6) | 0,56 |
| CDV3 | CD521865.1 | agencourt_14355147 nih_mgc_191 cdna clone image:30413831 5' | 0,53 |
| RPL24 | BQ062869.1 | agencourt_6826789 nih_mgc_99 cdna clone image:5924410 5' | 0,52 |
| HSPA14 | NM_016299.1 | heat shock 70kda protein 14 (hspa14) | 0,52 |
| MGC40405 | AL833918.2 | mrna; cdna dkfzp564o052 (from clone dkfzp564o052) | 0,51 |
| HDDC2 | NM_016063.1 | chromosome 6 open reading frame 74 (c6orf74) | 0,51 |
| YIPF4 | NM_032312.2 | yip1 domain family, member 4 (yipf4) | 0,47 |
| CKAP1 | W89079.1 | zh70f04r1 soares_fetal_liver_spleen_1nfls_s1 cdna clone image:417439 5' similar to gb:z23102_rna1 dna-directed rna polymerase ii 14.2 kd polypeptide (human) | 0,46 |
| PPIL5 | BX648029.1 | mrna; cdna dkfzp686j1525 (from clone dkfzp686j1525) | 0,45 |
| CYP19A1 | BX102808.1 | soares fetal liver spleen 1nfls cdna clone imagp998b18114 | 0,44 |
| BM670971.1 | BM670971.1 | ui-e-dw1-ahe-d-15-0-uis1 ui-e-dw1 cdna clone ui-e-dw1-ahe-d-15-0-ui 3' | 0,42 |
| NSUN4 | W90137.1 | zh75c06s1 soares_fetal_liver_spleen_1nfls_s1 cdna clone image:417898 3' | 0,42 |
| NDUFS8 | NM_002496.1 | nadh dehydrogenase (ubiquinone) fe-s protein 8, 23kda (nadh-coenzyme q reductase) (ndufs8) | 0,40 |
| TREH | NM_007180.1 | trehalase (brush-border membrane glycoprotein) (treh) | 0,39 |
| EME1 | BX405101.2 | t cells (jurkat cell line) cdna clone cs0dh001yc11 3-prime | 0,35 |
| WFDC12 | NM_080869.1 | wap four-disulfide core domain 12 (wfdc12) | 0,31 |
