# Supplementary material for: Expression Profiling of Rectal Tumors Defines Response to Neoadjuvant Treatment Related Genes
Source: PLoS One. 2014 Nov 7;9(11):e112189. doi: 10.1371/journal.pone.0112189 (PMC4224421; doi:10.1371/journal.pone.0112189)
Supplement: Table S3 — Area under curve (AUC) value and 95% confidence interval (CI) were calculated to determine the specificity and sensitivity of response to treatment prediction. (DOCX) [file pone.0112189.s005.docx]

|  | **Gene Name** | **AUC** | **Cut-off** |  |
| --- | --- | --- | --- | --- |
| **Microarray´data** | **Gng4** | 0.750 | 5.59 | Sensitivity: 70% |
|  |  |  |  | Specificity: 81.3% |
|  | **c-Myc** | 0.862 | 64.45 | Sensitivity: 70% |
|  |  |  |  | Specificity: 100% |
|  | **Pola1** | 0.850 | 167.74 | Sensitivity: 60% |
|  |  |  |  | Specificity: 75% |
|  | **Rrm1** | 0.806 | 5.52 | Sensitivity: 60% |
|  |  |  |  | Specificity: 57% |
